# Exercise echocardiography for improved assessment of diastolic filling dynamics

**DOI:** 10.1113/EP092177

**Published:** 2025-02-09

**Authors:** Mads Fischer, Thomas Bonne, Magnus Bak Klaris, Emil Lenzing, Eric J. Stöhr, Jacob Bejder, Carsten Lundby, Nikolai B. Nordsborg, Lars Nybo

**Affiliations:** ^1^ Department of Nutrition, Exercise and Sports University of Copenhagen Copenhagen Denmark; ^2^ Institute of Sport Science Leibniz University Hannover Hannover Germany; ^3^ Faculty of Social and Health Sciences Section for Health and Exercise Physiology Campus Lillehammer Inland University of Applied Sciences Lillehammer Norway

**Keywords:** cardiorespiratory fitness, echocardiography, endurance, exercise, maximal oxygen uptake, myocardial function, stroke volume

## Abstract

During exercise stress, heart rate (HR) increases to support cardiac output, which also reduces ventricular filling time. Although echocardiography is widely used to assess cardiac function, studies display conflicting data on the dynamic changes in the healthy trained and untrained heart during rest and acute exercise stress. To address these discrepancies, we tested a new echocardiography exercise protocol on two groups with significant differences in cardiorespiratory fitness. Ten untrained individuals with maximal oxygen uptake of 38 ± 8 ml/kg/min and 10 endurance‐trained athletes matched for body surface area but with higher maximal oxygen uptake (71 ± 5 ml/kg/min) were evaluated at rest, during semi‐recumbent cycling at 25 and 75 W and at a relative workload intensity eliciting a HR of 140 beats/min (HR140). Stroke volume was 36% higher in the trained at rest, and this difference increased during exercise to 42% at 25 W, 46% at 75 W and 63% at HR140 (all *P* < 0.05). In contrast, no group differences were found in markers of myocardial function (ventricular contraction and relaxation velocities) or other traditional echocardiographic measures of ventricular function at rest or exercise for a given HR. However, while similar at rest, diastolic and systolic function provided limited insight into differences between less fit and highly fit individuals. The new exercise echocardiography protocol improves the ability to uncover differences in dynamic changes in diastolic filling capacity that explain the previously reported higher end‐diastolic compliance in endurance‐trained athletes.

## Abstract (highlights)

- **What is the central question of this study?**
  Do dynamic changes in cardiac function during acute exercise stress differ between healthy untrained and endurance‐trained individuals, and does exercise echocardiography improve detection of potential differences?
- **What is the main finding and its importance?**
  Endurance‐trained athletes exhibit significantly higher stroke volumes and diastolic filling rates during exercise compared with untrained individuals, despite similar echocardiographic markers when matched for heart rate. Our study shows that conventional echocardiographic measures are insufficient to detect these functional differences. The key finding lies in the ability of the new exercise echocardiography protocol to reveal enhanced diastolic filling capacity in athletes, which explains the higher end‐diastolic compliance observed in endurance‐trained individuals.

## 1 INTRODUCTION

The ability to elevate cardiac output is essential, both for the ability to complete everyday physical activities and for exercise performance dependent on arterial oxygen delivery, because it is a major determinant for maximal oxygen uptake (${\dot{V}}_{O_{2}\max}$), a hallmark for cardiorespiratory fitness (Calbet et al., 2004). From a health perspective, the ability to increase cardiac output is also fundamental for coping with stressors such as environmental heat stress and a range of diseases (Calbet et al., 2004; Fischer et al., 2022; Giovannini et al., 1983; Rowell et al., 1969). Accordingly, for diagnostic purposes and for research studies involving healthy participants, it is highly relevant to be able to identify parameters that represent enhanced cardiac filling during stressful conditions.

Echocardiography has a very long tradition in both clinical settings and exercise studies, where it is amongst the most common cardiac imaging methods applied. The recent technological and methodological advancements in echocardiography in resting, exercise and pharmacological stress conditions have improved our knowledge on the assessment of both cardiomyopathies and the adaptations occurring with exercise training (Cooke et al., 2018; Dumanoir et al., 2007; Flanagan et al., 2023; Pelliccia et al., 2021; Pellikka et al., 2020). Despite this, it is questionable whether traditional echocardiographic variables, such as left ventricular (LV) longitudinal strain, twist, peak mitral valve (MV) early diastolic filling (E) and peak MV annular systolic (s′) and diastolic (e′) velocity, are adequate for detecting functional differences between less fit and highly fit individuals as evaluated in both cross‐sectional and longitudinal studies (Arbab‐Zadeh et al., 2014; Baggish et al., 2008; Bhella et al., 2014; Egelund et al., 2017; Fujimoto et al., 2010; Levine, 2008; Nio et al., 2020).

For example, Cooke et al. (2018) reported that the greater stroke volume in endurance athletes compared with less fit individuals was not picked up by standard echocardiographic measures of LV mechanics during rest. Furthermore, LV twist, torsion and twist‐to‐shortening velocities were similar during rest across the two groups of healthy participants despite vast difference in peak oxygen uptake. The study revealed clear between group changes from rest to exercise, and additional alterations were induced by superimposed hypoxia. However, differences appeared to be dictated by changes in heart rate rather than group dissimilarities in parameters indicative of diastolic or systolic function (Cooke et al., 2018). These observations also make it unclear whether adaptations in echocardiographic measures of cardiac function following training intervention studies are driven mainly by changes in heart rate and the derived effects on diastolic filling time or are truly reflective of changes in ventricular function (Macnamara et al., 2021; Quintana et al., 2005). Furthermore, studies dedicated to investigating differences between trained athletes and less fit (untrained) individuals provide conflicting results, because some studies (George et al., 2010) report a higher MV early filling velocity and elevated e′ in athletes during rest, whereas other studies report the reverse (i.e. lower values in endurance athletes compared to less fit individuals) (Brown et al., 2020; Flanagan et al., 2023; George et al., 2010).

The contrasting results in markers of diastolic function, shown by varying findings of e′, might relate to the inherent bias in assessing cardiac function at rest. Many echocardiographic markers, such as e′, MV E and s′, are affected by changes in preload, afterload, inotropic factors and heart rate during rest and exercise (Chant et al., 2018; Munch et al., 2014). For example, the heart rate of highly fit individuals is consistently lower both at rest and during exercise with a fixed workload affecting the duration of both the diastole and systole. As reported by Cooke et al. (2018) and Howden et al. (2018), these discrepancies persist when assessing cardiac function during exercise stress, because many studies prescribe the stress relative to the maximal capacity of individuals (i.e. a percentage of their maximal/peak oxygen uptake or work intensity), rather than intensities matched to fixed workloads, heart rate and/or body size as is done with most resting parameters of cardiac function and morphology. Although using relative intensities, such as percentage maximal aerobic power, can facilitate comparisons by normalizing to exercise capacity, this method could obscure differences in cardiac function. This is because the workload is adjusted to each individual rather than reflecting differences in cardiac capacity that are normalized to total body mass, similar to practices in evaluations of maximal oxygen uptake (${\dot{V}}_{O_{2}\max}$) (Clausen et al., 2018; Howden et al., 2018; Kodama, 2009; Levine, 2008).

To overcome the likely masking of cardiac adaptation to long‐term training, we developed a combined rest–exercise echocardiographic protocol. This protocol enables examination of the transition from rest to exercise and it allows for comparisons of cardiac responses in two conditions: a consistent absolute workload and a relative workload adjusted to elicit a target heart rate of 140 beats/min (HR140). We then applied the protocol to untrained individuals and endurance‐trained athletes matched for body size [body surface area (BSA)] to evaluate parameters of relevance for differences in functional capacity.

We hypothesized that evaluations of cardiac function based only on resting echocardiography would limit the ability to detect functional differences between untrained and highly trained participants. In contrast, we hypothesized that using the combined rest–exercise echocardiographic protocol in groups with vast differences in cardiorespiratory fitness would allow us to identify echocardiographic parameters of particular importance for functional capacity.

## 2 MATERIALS AND METHODS

### 2.1 Ethical approval

In accordance with the *Declaration of Helsinki*, the study received approval from the Ethics Committee of Copenhagen and Frederiksberg communities (H‐21011041). The study was registered as a clinical trial at clinicaltrials.gov (NCT05191979), and prior to participation in the study, written informed consent was obtained from all participants, affirming their voluntary enrolment.

### 2.2 Participants

Ten elite endurance‐trained athletes (10 male cyclists, trained group) and 10 healthy, but markedly less fit participants (five males and five females; here referred to as the untrained group because they were not engaged in any formalized training before testing), free of any known diseases, participated in the study. All participants declared not to have donated blood within the last 3 months, and the untrained volunteers declared not to have participated in regular or organized physical training within the past 1 year. The untrained participants included here were tested before commencing a larger 1 year training study including participants of both biological sexes, because comparing functional parameters measured by echocardiography appears to be a matter of size and training status, not biological sex (Baggish et al., 2008; Lang et al., 2015). Inclusion criteria were ${\dot{V}}_{O_{2}\max}$ of >55 ml/min/kg for the trained men and <45 mL/min/kg for the untrained men and women, age between 18 and 45 years, not under medical treatment, absence of metabolic, chronic diseases, uncontrolled arrhythmia, second or third degree atrioventricular block or sick sinus syndrome assessed from a standard 12‐lead ECG, no history of smoking or alcohol consumption (≥14 items/week) and with a decent acoustic window for echocardiography.

### 2.3 Experimental protocol

Experimental procedures were conducted over the course of 2 days for the untrained subjects, allowing them to be optimally recovered for the exercise tests. The trained group performed all the tests on the same day.

Before arriving at the laboratory, participants were instructed to avoid caffeinated drinks for 12 h. If arriving before midday, they were instructed to have their usual breakfast and drink ≥500 mL of water. The hydration status of the participants was evaluated using urine specific gravity (refractometer; Atago Inc., Bellevue, WA, USA) to ensure they were hydrated before the test (verified by urine specific gravity < 1.025; Cheuvront et al., 2010).

After the collection of urine samples and anthropometric measurements, participants were prepared for the study. ECG electrodes and a brachial blood pressure cuff were attached while the participants lay supine with raised legs for 10 min (see section 2.4.2). Following this rest period, a blood sample was collected (see section 2.4.6), the legs were lowered for 5 min, and blood pressure was measured before a standard resting echocardiographic assessment was initiated (Lang et al., 2015).

After the echocardiographic stress test, trained participants proceeded to aerobic capacity testing on a upright bike ergometer (see section 2.4.4). Untrained participants conducted the aerobic capacity test 2 days before the echocardiographic examination. For the untrained subjects, the protocol began with a warm‐up phase involving incremental submaximal intensities. After a brief rest period of <5 min, ${\dot{V}}_{O_{2}\max}$ was assessed during an incremental exercise test. The initial workload was set at 75 W for females and 90 W for males. The workload increased by 25 W/min, until the point of volitional fatigue. For trained subjects, the test protocol initiated with a 3 min resting phase on the bike, followed by a 5 min warm‐up phase at 100 W. The resistance gradually increased, starting from 100 W and continuing with intensity increments of 30 W/min until participants reached the point of exhaustion.

To determine the ${\dot{V}}_{O_{2}\max}$, participants were required to fulfil at least two out the following three criteria: a plateau in oxygen uptake (${\dot{V}}_{O_{2}}$) despite escalating workload; a respiratory exchange ratio surpassing 1.1; and a heart rate surpassing 90% of their age‐predicted maximal heart rate (estimated in accordance with Farazdaghi & Wohlfart, 2001; Wohlfart & Farazdaghi, 2003).

### 2.4 Instrumentation and measurements

#### 2.4.1 Anthropometrics and blood/urine measurements

Hydration status was measured by urine specific gravity. Body mass was assessed with a platform scale (InBody 270; Cerritos, CA, USA). Body surface area was calculated using the DuBois & DuBois formula in order to obtain BSA‐normalized values (Du Bois, 1916): $\mathrm{BSA}\left(m^{2}\right)=0.202\times {\left(\mathrm{body}\mathrm{mass}\right)}^{0.425}\times {\left(\mathrm{height}\right)}^{0.725}$.

Blood haemoglobin concentrations were analysed using the ABL800 FLEX analyser (Radiometer, Bronshoj, Denmark). Additionally, haematocrit was determined by subjecting blood samples to centrifugation at 16,058 *g* for 4 min (Hermle Z 207H, Germany).

#### 2.4.2 Heart rate and blood pressure

Heart rate was recorded using a three‐lead ECG (GE Medical Systems, Madison, WI, USA). Resting brachial systolic and diastolic blood pressures were measured using a brachial sphygmomanometer (M7; OMRON, Vernon Hills, IL, USA). Continuous mean arterial pressure was measured using a finger pressure cuff (Human NIBP Nano System, ADInstruments, Bella Vista, NSW, Australia) placed on the middle phalanx of the middle finger. As described previously (Fischer et al., 2022), given that finger pressure‐derived blood pressures have been reported to underestimate those by brachial sphygmomanometer (Bos et al., 1996), a correction factor was determined as the ratio of mean arterial pressure measured by finger pressure cuff at baseline to that by the brachial sphygmomanometer. The heart rate was recorded using the Powerlab 16/35 data acquisition system and LabChart 8 software (ADInstruments).

#### 2.4.3 Cardiac morphology and function

Transthoracic echocardiography was conducted using a GE Vivid E9 ultrasound machine (GE Healthcare) equipped with a 2.5 MHz transducer. Participants were positioned in a supine position and turned onto their left side in an air‐conditioned and darkened room maintained at 23°C. The echocardiographic examinations were conducted by an experienced echocardiographer (M.F.) trained at the Department of Cardiology, Copenhagen University Hospital ‘Rigshospitalet’, Denmark, and the data were stored offline and analysed using EchoPAC (GE Healthcare, v.203). The resting echocardiographic examination adhered to the current guidelines from the American Society of Echocardiography and the European Association of Cardiovascular Imaging (Lang et al., 2015).

Pulsed‐wave (PW) tissue Doppler imaging (TDI) was used to measure mitral annular peak velocities during the systolic (s′), early diastolic (e′) and late diastolic (a′) phases by averaging the values obtained from the septal and lateral mitral annulus. The LV mass was calculated using the cube formula based on two‐dimensional measurements (Lang et al., 2015). The frame rate was set at a minimum of 57 frames/s during rest and 89 frames/s during exercise to ensure optimal spatial resolution. Stroke volume was evaluated during rest and exercise using the velocity–time integral (VTI) method (LVOT × VTI), with the measurement of the LV outflow tract diameter at diastole (LVOT) recorded at rest, and was assumed to remain constant (Rassi et al., 1988). In order to calculate LV filling rates, the diastolic duration was calculated by subtracting the estimated ejection time (Moran et al., 1995) from the R–R interval duration. Mean diastolic filling rates were then calculated by dividing stroke volume by the diastolic duration (SV/td), as reported by others (Ferguson et al., 2001; Levy et al., 1993).

To minimize analytical variability, all examinations were analysed offline with a minimum of three cardiac cycles, and semi‐automatic tracking was used for accurate Doppler and B‐mode measurements. Speckle tracking analysis of strain and twist have been described previously (Stöhr et al., 2011). In short, regions of interest were traced semi‐automatically along the endocardial borders in each view, and speckles within the region of interest were tracked automatically throughout the cardiac cycle for three consecutive cycles.

#### 2.4.4 Aerobic capacity test

During exercise testing, expired gases and volumes were measured using open‐circuit spirometry to determine rates of ${\dot{V}}_{O_{2}}$ and carbon dioxide production. Two systems were used for practical reasons: untrained participants were tested using the Quark CPET (COSMED, Rome, Italy) on a bike ergometer (Excalibur Sport, Lode BV), whereas trained participants were assessed using the Vyntus™ CPX system (Vyaire Medical) tested on the subjects’ own bikes mounted on a direct drive hometrainer (Tacx Neo 2T T2875, Garmin, Olathe, KS, USA). Results obtained from the Vyntus™ CPX system were tested and adjusted based on calibration tests using a metabolic calibrator (VacuMed, Ventura, CA, USA), ensuring consistent measurements across the two systems.

#### 2.4.5 Exercise stress echocardiography

During the echocardiographic exercise stress test, the participants cycled on a semi‐recumbent tilting cycle ergometer (eBike EL, GE Healthcare), and echocardiographic measures were recorded. The participants completed three consecutive 10 min intervals at two absolute workloads of 25 and 75 W and at a relative workload eliciting a heart rate of ∼140 beats/min (HR140). The Subjects were instructed to maintain a cadence of 55–65 r.p.m. throughout the 30 min protocol and tolerated the 5 min stages without left lateral tilt without complaints. However, during the final 5 min with left lateral tilt, they reported general discomfort owing to the awkward position, but no pain or unusual exercise‐related discomfort was expressed. The final relative workload successfully normalized heart rates between groups (*P* > 0.1), yielding 144 ± 10 beats/min in the untrained group and 137 ± 8 beats/min in the trained group. Owing to poor comfort while tilted, each interval began with 5 min of cycling in a semi‐recumbent position, followed by 5 min of semi‐recumbent cycling with a 45° left lateral tilt while the operator recorded the echocardiographic images.

#### 2.4.6 Intravascular blood volume

Using an automated carbon monoxide rebreathing system (Detalo Performance™; Detalo Instruments, Denmark) and following the previously described method (Siebenmann et al., 2017), we conducted measurements and calculations to determine the total intravascular blood volume, plasma volume and haemoglobin mass.

### 2.5 Statistical analysis

**FIGURE 1:**
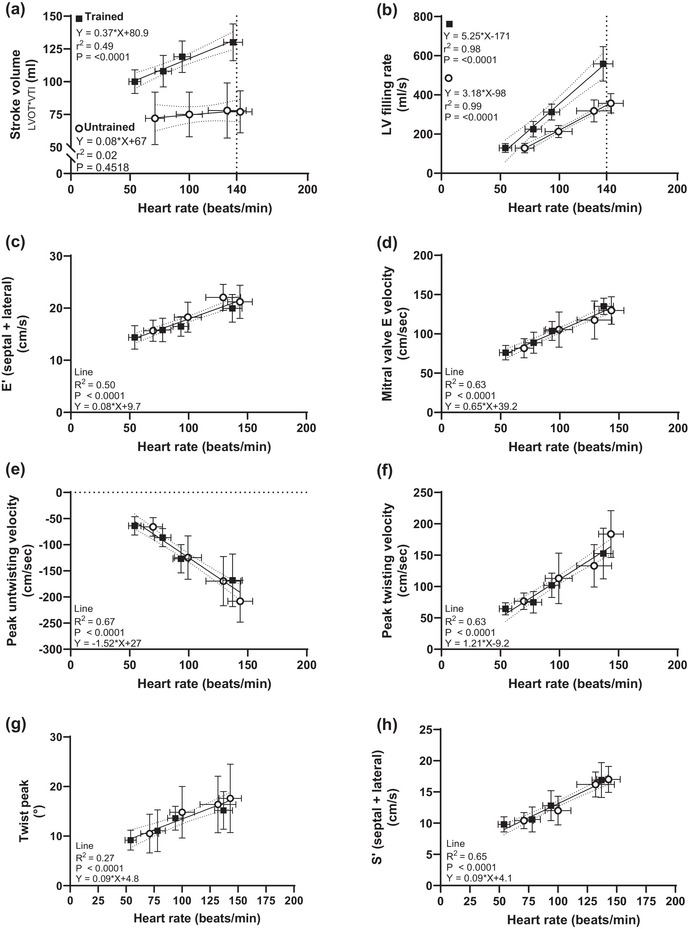
Relationships between heart rate (in beats per minute) and stroke volume (a), left ventricular (LV) filling rate (b), Pulsed wave tissue doppler imaging peak early‐diastolic LV lateral wall tissue velocity (E′) (c), mitral valve early‐diastolic LV filling velocity (d), LV ejection fraction (e), Peak twisting velocity (f) and peak untwisting velocity (g). Open circles indicate untrained; squares indicate trained. When individual (group‐specific) regressions did not differ significantly, regressions were calculated based on data for both groups. Regression lines are shown in c–h with trends for all data (i.e. combined group trends) with their 95% confidence interval. Regression lines in a and b show the trend of each group with their 95% confidence interval. Mean values are presented as the mean ± SD.

**FIGURE 2:**
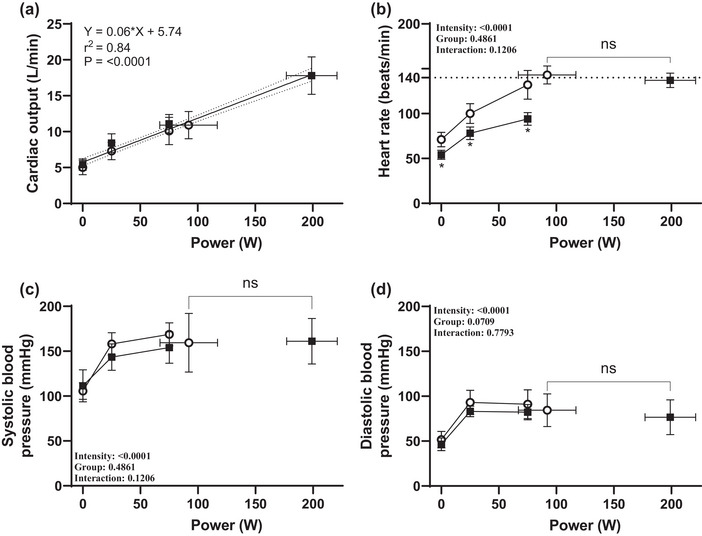
Relationships between power output (in watts) and stroke volume (a), heart rate (b), systolic blood pressure (c) and diastolic blood pressure (d). Data for fixed load exercise (b–d) were analysed via a mixed‐effects model with the independent factors of exercise intensity and group. Data for fixed load exercise (b–d) were analysed using Student's *t*‐test. Open circles indicate untrained; squares indicate trained. Regression lines (a) show the trend of all data (i.e. combined group trends) with their 95% confidence interval. Values are the mean ± SD.

Student's unpaired *t*‐tests were used to compare non‐repeated variables, such as age, body weight, blood volume and ${\dot{V}}_{O_{2}\max}$, in the subject characteristics analysis (Tables 1 and 2). The data were analysed using a mixed‐effects model with the independent factors of condition (baseline, 25 W and 75 W; repeated variable) and group (trained or untrained; non‐repeated variable) (Tables 2 and 3). Correlation analysis (Figures 1 and 2) was conducted using Pearson's methods, and when group‐specific regression lines did not differ significantly regressions were calculated based on the combined data. Time‐dependent data, including mean arterial pressure and heart rate, were analysed using 4 min averages at the end of each data collection period (e.g., baseline, 25 W, 75 W and HR140). *Post hoc* multiple comparisons were performed with a Bonferroni correction. Statistical analyses were conducted using Prism software (GraphPad Prism v.9.1.1, GraphPad, San Diego, CA, USA). The results are reported as the mean ± SD, and a significance level of α < 0.05 was used.

**TABLE 1:** Characteristics of untrained and trained subjects.

Subject characteristics	Untrained	Trained	*P*‐value
Age (years)	27 ± 6	23 ± 3	0.0736
Height (cm)	177 ± 9	185 ± 6	**0.0284**
Body mass (kg)	73.1 ± 13.2	71.4 ± 5.1	0.7133
Body surface area (m^2^)	1.89 ± 0.2	1.93 ± 0.1	0.4947
Fat‐free body mass (kg)	51.7 ± 13.1	62.5 ± 4.2	0.0923
Fat percentage (%)	26 ± 6	13 ± 4	**<0.0001**
Total blood volume (L)	4.6 ± 1.1	6.6 ± 0.3	**<0.0001**
Systolic blood pressure (mmHg)	120 ± 9	123 ± 10	0.5951
Diastolic blood pressure (mmHg)	74 ± 7	65 ± 5	**0.0066**
${\dot{V}}_{O_{2}\max}$ (L/min)	2.8 ± 0.9	5.0 ± 0.4	**<0.0001**
${\dot{V}}_{O_{2}\max}$ (ml/kg/min)	37.8 ± 7.6	70.7 ± 5.0	**<0.0001**
Power at HR140 (W)	93 ± 23	199 ± 22	**<0.0001**

*Note*: Values are the mean ± SD. Significant *P*‐values are shown in bold. Abbreviation: HR140, relative workload eliciting a heart rate of ∼140 beats/min; ${\dot{V}}_{O_{2}\max}$, maximal pulmonary oxygen consumption.

**TABLE 2:** Baseline left ventricular morphology and function in untrained and trained subjects.

Left ventricular morphology and function at rest	Untrained	Trained	*P*‐value
LV septal thickness at end diastole (mm)	9 ± 2	12 ± 1	**0.0004**
LV posterior wall at end diastole (mm)	8 ± 1	10 ± 1	**0.0007**
LV mass (g)	138 ± 54	253 ± 32	**<0.0001**
LV mass index BSA (g/m^2^)	72 ± 22	133 ± 13	**<0.0001**
LV end‐diastolic volume BP Simpson's (ml)	92 ± 27	161 ± 13	**<0.0001**
LV end‐systolic volume BP Simpson's (ml)	41 ± 13	79 ± 11	**<0.0001**
LV ejection fraction BP Simpson's (%)	56 ± 3	51.1 ± 3.8	**0.0041**
LVOT diameter (mm)	19.3 ± 2.9	23.8 ± 1.2	**0.0002**
E/A ratio	1.9 ± 0.4	2.1 ± 0.3	0.1574
Global longitudinal strain (%)	−18.2 ± 1.1	−16.9 ± 2.4	0.1273
Left atrial end‐diastolic volume (ml)	49 ± 16	70 ± 12	**0.0066**
Right atrial end‐diastolic volume (ml)	43 ± 12	71 ± 16	**0.0003**

*Note*: Values are the mean ± SD. Significant *P*‐values are shown in bold. Abbreviations: BP Simpson's, biplane Simpson's method; BSA, body surface area; E/A, early (E) and late (A) diastolic transmitral peak flow velocity; LV, left ventricle; LVOT, Left ventricular outflow track.

## 3 RESULTS

### 3.1 Anthropometrics, cardiac dimensions and physical fitness

In terms of body anthropometrics, the two groups displayed similar age, body mass and BSA (all *P* > 0.05), whereas absolute and relative (weight‐adjusted) maximal aerobic capacity (${\dot{V}}_{O_{2}\max}$ and ${\dot{V}}_{O_{2}\max}$/kg) was 79% and 87% higher, respectively, in the trained group compared with the untrained group. The trained group also had a 44% higher blood volume (corresponding to a difference of ∼2 L; *P* < 0.001), while their body fat percentage was lower (see Table 1).

Furthermore, due to both greater septal and posterior wall thickness, in addition to larger LV end‐diastolic diameter, absolute and relative LV mass was noticeably higher in the trained compared with untrained individuals during rest (see Table 2). The LV outflow tract diameter of the trained group was larger than that of the untrained group (*P *< 0.05) but similar to that of size‐matched former professional cyclists (*P *> 0.35) (Valenzuela et al., 2023). Systolic blood pressure was similar and normal (∼120 mmHg) in both groups, whereas the trained group had a lower resting diastolic pressure (see Table 1).

### 3.2 Measures of cardiac function

Stroke volume was 36% higher in the trained group at rest, and this difference increased during exercise to 42% at 25 W, 46% at 75 W and 63% at HR140 (all *P* < 0.05; see Figure 1). For all conditions, the higher stroke volume related to increased end‐diastolic dimensions in the trained group. In contrast, neither enhanced emptying, as evaluated by lower end‐systolic dimensions, nor differences in fractional shortening contributed to the superior stroke volume at rest or exercise in the trained subjects.

At rest, the higher stroke volume and enhanced filling in the trained group related exclusively to a longer duration of diastole (allowed by the lower resting heart rate), while the diastolic LV filling rate was similar across groups. In contrast, during exercise the trained group had a pronounced increase in LV filling rate (∼3‐fold higher at HR140 compared with rest), while the change was more modest in the untrained group (∼2‐fold increase from rest to exercise at HR140; see Figure 1b). The enhanced exercise‐induced diastolic function in the trained group did not translate into differences in echocardiographic variables such as LV untwisting rate, peak mitral valve early diastolic filling (E), peak MV annular systolic (s′) or diastolic (e′) velocity when compared for a given heart rate (see Figure 1c–h).

## 4 DISCUSSION

The present findings provide a potential future approach for using echocardiography to explore how endurance training affects the healthy heart in terms of improved filling properties. In accordance with our first hypothesis, group differences were modest, small or, for some parameters, absent at rest but differentiated during the exercise protocol. This is most clearly exemplified by similar diastolic filling rates at rest, but noticeable differences during exercise that allowed for enhanced diastolic filling and elevated exercise stroke volume in the trained group. Our data also demonstrate that a range of echocardiographic parameters (measures of deformation, twisting, contraction or relaxation velocities) that might be useful for exploring functional deficiencies in pathophysiological conditions are not sensitive for evaluation of training effects in the healthy heart because they were similar across groups. Furthermore, changes in these factors during exercise seemed to be dictated by heart rate rather than functional differences in volume flow and myocyte contraction/relaxation velocities. The latter could very well explain some paradoxical observations in cross‐sectional and longitudinal exercise studies, because a lower heart rate at rest or during evaluations at a given (absolute work) intensity will reduce factors such as s′, ejection fraction and measures of diastolic filling (e.g., MV E and e′). Such findings would incorrectly suggest inferior function rather than improved reserve of the functions herein following exercise training.

### 4.1 Benefits from a combined rest–exercise protocol

Training effects on cardiac function are in the majority of studies assessed with echocardiography in resting conditions, where autonomic balance and heart rate may co‐vary with the training status, whereas factors such as preload and afterload (pulmonary wedge and arterial pressures) typically do not differ in healthy subjects despite different training status (Valenzuela et al., 2023; Proctor et al., 1998; Bada et al., 2012). However, as exercise intensity increases, both filling pressure and arterial pressure rise, while cardiac filling and emptying times decrease owing to an accelerated heart rate. These changes significantly alter the haemodynamics that affect cardiac function (Munch et al., 2014; Chant et al., 2018). Therefore, scaling exercise stress to normative values adjusted for heart rate might prove useful to distinguishing normal physiological responses from pathological ones, irrespective of fitness levels.

In the present study, there was no interaction between group and absolute workloads for cardiac output (Table 2), which is in alignment with the findings of several other studies (Calbet et al., 2004; Proctor et al., 1998; Bada et al., 2012). The functional measures demonstrating an interaction between groups were correlated strongly with heart rate (Figure 1), and the assessment of filling rates and stroke volume revealed a significantly steeper slope for the trained group (Figure 1a, b). Peak inflow, contraction and relaxation rates did not elicit any differences when assessed during relative workloads (Figure 1c–h), but during absolute load a significant interaction was found in many measures of systolic and diastolic function, with higher rates of filling, contraction and relaxation found in the untrained (Table 3). The finding of a significant correlation between heart rate and parameters of LV mechanics is noteworthy, especially given that this relationship has not been explored in prior studies. This finding suggests that previous conclusions regarding altered twist mechanics (Figure 1e–g) could be confounded by inherent differences or changes in secondary factors, such as preload and heart rate (Fu et al., 2004; Stöhr et al., 2011). These findings underscore the importance of using both relative and absolute workloads for a comprehensive assessment of cardiac function. Moreover, this approach allows for the identification of heart rate‐dependent measures, enabling the examination of a dose–response relationship.

**TABLE 3:** Echocardiographic variables at baseline and during exercise stress testing at absolute work intensities.

Left ventricle Function	Exercise intensity				*P*‐value		
		Rest	25 W	75 W	Intensity	Group	Interaction
Internal dimension diastole (cm)	Untrained	4.8 ± 0.6	4.8 ± 0.6	4.7 ± 0.6	0.4032	**0.0001**	**0.0068**
	Trained	5.6 ± 0.4^a^	5.8 ± 0.4^b^	5.8 ± 0.4^b^			
Internal dimension systole (cm)	Untrained	3.4 ± 0.3	3.3 ± 0.5	3.1 ± 0.5	0.5921	**0.0005**	**0.0001**
	Trained	4.1 ± 0.4^a^	4.3 ± 0.5^b^	4.0 ± 0.4^b^			
Fractional shortening (%)	Untrained	29 ± 5	30 ± 5	34 ± 6	**<0.0001**	0.0682	0.3535
	Trained	27 ± 3	26 ± 5	31 ± 3			
LVOT VTI (cm)	Untrained	24.3 ± 3.1	25.4 ± 3.3	27.2 ± 4.2	**<0.0001**	0.2757	0.2673
	Trained	22.4 ± 2.1	24.3 ± 2.4	26.6 ± 1.8			
Stroke volume (LVOT × VTI) (ml)	Untrained	72 ± 20	74 ± 17	79 ± 20	**<0.0001**	**<0.0001**	**0.0495**
	Trained	100 ± 9^a^	108 ± 12^b^	119 ± 12^b^			

Diastolic function							
Mitral valve E velocity (cm/s)	Untrained	81.0 ± 11.9	105 ± 21	118 ± 23	**<0.0001**	**0.0405**	0.3315
	Trained	76.1 ± 9.1	89 ± 13^a^	104 ± 12			
PW TDI E′ (cm/s)	Untrained	15.8 ± 1.9	18.2 ± 2.7	22.8 ± 3.2	**<0.0001**	**0.0035**	**0.0016**
	Trained	14.4 ± 2.3	15.8 ± 2.3	16.5 ± 1.9^b^			
E/E′ ratio	Untrained	5.2 ± 1.1	5.8 ± 0.7	5.2 ± 1	0.8360	0.1691	**0.0018**
	Trained	5.4 ± 0.9	5.7 ± 1.2	6.3 ± 0.9^a^			
Untwisting velocity peak (cm/s)	Untrained	−66 ± 18	−125 ± 41	−170 ± 47	**<0.0001**	**0.0076**	**0.0420**
	Trained	−64 ± 18	−86 ± 17	−127 ± 27			
Systolic function							
PW TDI S′ (cm/s)	Untrained	10.5 ± 1.3	12.2 ± 2.2	16.9 ± 2.6	**<0.0001**	**0.0016**	**0.0014**
	Trained	9.8 ± 1.2	10.6 ± 2	12.8 ± 2.4^b^			
Twist peak (°)	Untrained	10.5 ± 3.9	14.8 ± 5.2	16.4 ± 5.7	**0.0007**	**0.0243**	0.4456
	Trained	9.2 ± 2	10.7 ± 2.9	13.6 ± 2.4			
Twist velocity peak (cm/s)	Untrained	76 ± 13	113 ± 40	133 ± 34	**<0.0001**	**0.0015**	0.1709
	Trained	64 ± 10	75 ± 17	102 ± 19			

*Note*: Data were analysed via a mixed‐effects model with the independent factors of exercise intensity and group. Values are the mean ± SD. Significant *P*‐values are shown in bold. Abbreviations: E/e, Early diastolic mitral inflow velocity to mitral annular velocity; LVOT, Left ventricular outflow tract; PW‐TDI, Pulsed‐Wave Tissue Doppler Imaging, VTI, velocity time integral.

^a^ *P *< 0.05 versus untrained.

^b^ *P *< 0.001.

### 4.2 Functional versus structural differences

Cross‐sectional studies suggest that resting measurements, specifically LV mass and volume, serve as effective parameters to differentiate between untrained and trained individuals (Boraita et al., 2022; La Gerche et al., 2012). However, for tracking longitudinal changes and understanding the underlying mechanisms of improved ventricular function, exercise evaluations appear indispensable.

For instance, Baggish et al. (2008) report a strong correlation between increases in both LV mass and early diastolic relaxation velocity (e′) following a 90 day retraining intervention (Baggish et al., 2008). In contrast, Cooke et al. (2018) and Fischer et al. (2025), among others, reported that measures such as MV E and e′ tend to remain unchanged or even decrease in trained individuals and with intensified training, whether evaluated at rest or with a matched work intensity (Brown et al., 2020; Cooke et al., 2018; Egelund et al., 2017; Fischer et al., 2025; Parsons et al., 2020). This discrepancy highlights a complex effect of chronic adaptations to exercise training on cardiac function, suggesting that to capture the dynamics of physiological changes over time fully, a combination of static and functional assessments is crucial. Our findings offer insights into this apparent paradox, indicating the need for a multifaceted approach to assess the impact of training on cardiac structure and function accurately.

Although the trained group displayed moderately higher stroke volume at rest compared with the untrained, they also exhibited a marked increase during exercise, whereas this exercise effect was absent in the untrained group (see Figure 1). Differences in diastolic filling rates and derived effects on stroke volume therefore became very clear during exercise. Although some of the morphological adaptations achieved via years of exercise training in athlete group might allow for superior function, it would not be picked up by standard resting echocardiography (Pfaffenberger et al., 2013). This correlation, alongside the observed enhanced increases in stroke volume under exercise stress, underscores the complexity of interpreting markers of diastolic function. Despite the anticipated improvements in diastolic function associated with increased fitness and cardiac mass, our study finds markers of diastolic function to be similar or even lower in the trained group.

Taken together, these findings indicate that the healthy trained and untrained hearts display altered filling properties for any given heart rate, but similar cardiac systolic function when exposed to increased relative exercise intensities despite significant differences in cardiac morphology. When conventional echocardiographic measurements are taken during matched relative stress, such as at fixed heart rates, differences between the untrained heart and the heart of an athlete do not show enhanced LV function as assessed by standard measures. This indicates that none of the conventional markers is ideal for assessing functional differentiation. Our finding might provide a basis for further studies to develop more effective diagnostic tools that can accurately reflect the functional effects associated with exercise training. These findings might be most pertinent to researchers investigating the effects of the functional adaptations in the healthy and pathological cardiac adaptations that relate to a functional cardiac improvement/impairment.

### 4.3 Limitations

Given that the present study involves healthy participants exclusively, it is important not to project findings or conclusions to pathophysiological conditions. Specifically, our study does not address subjects with diseases that could affect ventricular filling or emptying, such as pulmonary or arterial hypertension, or the impact cardiac function attributable to ischaemia or similar conditions. The findings obtained from this study lay a foundational framework for understanding normal physiological responses to exercise. Moreover, these findings could serve as a reference for future research aimed at the cardiac pathophysiological response to exercise stress, thereby advancing the current understanding of cardiac diseases.

The exercise modality used in the present study and in others studies on stress echocardiography does not fully replicate the cardiovascular response associated with upright cycling (Beaumont et al., 2021). The semi‐supine position on an ‘echo‐bike’ alters gross efficiency significantly and, in particular, when combined with a left lateral tilt (Beaumont et al., 2021). This alteration might be more pronounced in individuals who are accustomed to a competitive cycling posture, including the trained participants in this study. However, no difference in cardiac output was observed between the groups, suggesting that the gross efficiency during this mode of cycling is similar for trained and untrained subjects. The maximal workload in this study was determined by using relative heart rate as a compromise to achieve the highest possible stroke volume (Munch et al., 2014; Stöhr et al., 2011). This methodology was selected to reduce the potential for hyperventilation and discomfort among participants which, at higher exercise intensities, could compromise the quality of the echocardiographic assessments.

The subjects were matched for BSA, which is the gold standard for cardiac morphological assessment. However, we acknowledge that BSA as an index might bias the interpretation of the findings because it does not account for lean body mass or blood volume, which have also been shown to provide stronger correlation with LV morphology in several studies (Carrick‐Ranson et al., 2013; Gomes et al., 2023). Therefore, the composition of the untrained group, which included an equal proportion of males and females, might influence the findings of this study. Previous research has demonstrated that males typically have a ∼8% larger LV mass compared with females, even after correcting for lean body mass (Gomes et al., 2023). Likewise, in our study the untrained male and female subjects showed an 8% difference in LV mass after indexing for lean body mass (Tables S1 and S2).

Cycling involves limited joint movements, and the difference in gross efficiency during fixed load intensities between trained and untrained individuals is insignificant (Mogensen et al., 2006). Therefore, using absolute workload allows for easy comparison between subjects, resulting in similar cardiac output (Figure 2). Although fixed loads are also used in clinical evaluations (Lancellotti et al., 2016), fixed intensity puts smaller or obese individuals at a disadvantage (Carrick‐Ranson et al., 2013), and the difference cannot be corrected by simply scaling to bodyweight, height or other calculated scaling adjustments (Chantler et al., 2005). Notably, although female subjects in the untrained group had the lowest BSA, they exhibited smaller stroke volumes than their male counterparts but showed no differences in echocardiographic measures of systolic (PW TDI S′) and diastolic (PW TDI E′) function.

## 5 CONCLUSION

Echocardiographic evaluations at rest were adequate for detecting differences between highly fit and less fit individuals in terms of gross functional measures, such as ventricular volumes and mass. However, evaluations during exercise are warranted to explore differences in diastolic filling capacity that characterize superior function in trained athletes, which is not identifiable at rest or via traditional measures of diastolic function.

## AUTHOR CONTRIBUTIONS

Mads Fischer and Lars Nybo conceptualized and designed the study. Data acquisition was performed by Mads Fischer, Thomas Bonne and Magnus Bak Klaris Data analysis and interpretation was performed by Mads Fischer, Thomas Bonne, Jacob Bejder, Emil Lenzing, Eric J. Stöhr, Carsten Lundby, Nikolai B. Nordsborg, Lars Nybo1 and Mads Fischer drafted the manuscript. All authors critically revised the manuscript, approved its final version and agree to be accountable for all aspects of the work in ensuring that questions related to the accuracy or integrity of any part of the work are appropriately investigated and resolved. All persons designated as authors qualify for authorship, and all those who qualify for authorship are listed.

## CONFLICT OF INTEREST

C.L. holds the position of Chief Executive Officer (CEO) at Detalo Health, Denmark, the company responsible for manufacturing the automated carbon monoxide rebreathing system, Detalo Performance™. The other authors have no conflicts of interest to disclose. The results of the study are presented clearly, honestly and without fabrication, falsification or inappropriate data manipulation.

## Supporting information




**TABLE S1** Characteristics of untrained male and female subjects. Abbreviations: FFM, fat‐free body mass; ${\dot{V}}_{O_{2}}$, pulmonary oxygen consumption; ${\dot{V}}_{O_{2}\max}$, maximal pulmonary oxygen consumption. Values are the mean ± SD.
**TABLE S2** Baseline left ventricular morphology and function in untrained male and female subjects. Abbreviations: BPS, biplane Simpsons method; BSA, body surface area; E/A, early (E) and late (A) diastolic transmitral peak flow velocity; LV, left ventricle; LVOT, left ventricular outflow track. Values are the mean ± SD.

## Data Availability

The datasets generated during and/or analysed during the present study are available from the corresponding author on reasonable request.
