# Supplementary material for: Exercise echocardiography for improved assessment of diastolic filling dynamics
Source: Exp Physiol. 2025 Feb 9;110(6):809–20. doi: 10.1113/EP092177 (PMC12128469; doi:10.1113/EP092177)
Supplement: Supplementary file 1 — TABLE S1 Characteristics of untrained male and female subjects. Abbreviations: FFM, fat‐free body mass; V˙O2, pulmonary oxygen consumption; V˙O2max, maximal pulmonary oxygen consumption. Values are the mean ± SD. TABLE S2 Baseline left ventricular morphology and function in untrained male and female subjects. Abbreviations: BPS, biplane Simpsons method; BSA, body surface area; E/A, early (E) and late (A) diastolic transmitral peak flow velocity; LV, left ventricle; LVOT, left ventricular outflow track. Values are the mean ± SD. [file EPH-110-809-s001.docx]

**SUPPLEMENTAL TABLES**

| **Subject Characteristics** | **Male** | **Female** | **P-value** |
| --- | --- | --- | --- |
| **Age (y)** | 26 ± 5 | 29 ± 7 | 0.4315 |
| **Height (cm)** | 1.81 ± 0.07 | 1.71 ± 0.11 | 0.1199 |
| **Body mass (kg)** | 83 ± 11 | 64 ± 3 | **0.0067** |
| **Body Surface Area (BSA) (m^2^)** | 2.03 ± 0.17 | 1.74 ± 0.11 | **0.0118** |
| **Fat free mass (FFM) (kg)** | 64.6 ± 8.9 | 49.1 ± 8.5 | **0.0232** |
| **Fat percentage (%)** | 22.2 ± 2.4 | 28.6 ± 13.6 | 0.9094 |
| **Total blood volume (L)** | 5.4 ± 1.1 | 3.9 ± 0.5 | **0.0195** |
| **Total blood volume index FFM (L/kg)** | 83 ± 7 | 80 ± 17 | 0.7785 |
| **Systolic (mm Hg)** | 128 ± 6 | 112 ± 2 | **0.0014** |
| **Diastolic (mm Hg)** | 73 ± 6 | 74 ± 9 | 0.8761 |
| **V̇_O2max_ (L/min)** | 3.4 ± 0.9 | 2.2 ± 0.3 | **0.0258** |
| **V̇_O2max_ (ml/kg/min)** | 40.2 ± 7.6 | 34.0 ± 5.7 | 0.1841 |
| **V̇_O2max_ index FFM (ml/kg/min)** | 52 ± 9 | 45 ± 10 | 0.2930 |
| **Power at HR140 (W)** | 112 ± 27 | 71 ± 11 | **0.0132** |

**Supplemental table 1** - Characteristics of untrained male and female subjects. *V̇*_O2_. Pulmonary oxygen consumption; *V̇*_O2max_. Maximal pulmonary oxygen consumption; FFM, fat-free body mass; Values are mean ± SD.

| **Left Ventricle morphology and function at rest** | **Male** | **Female** | **P-value** |
| --- | --- | --- | --- |
| **LV septal thickness at end-diastole (mm)** | 10 ± 1 | 8 ± 1 | 0.0943 |
| **LV posterior wall at end-diastole (mm)** | 8 ± 2 | 7 ± 1 | 0.1916 |
| **LV mass (g)** | 171 ± 58 | 104 ± 21 | **0.0416** |
| **LV mass index FFM (g/kg)** | 2.6 ± 0.7 | 2.2 ± 0.6 | 0.2918 |
| **LV mass index BSA (g/m^2^)** | 84 ± 24 | 60 ± 13 | 0.0879 |
| **LV end-diastolic volume BPS (ml)** | 109 ± 25 | 75 ± 15 | **0.0304** |
| **LV end-diastolic volume BPS index FFM (ml/kg)** | 54 ± 11 | 43 ± 8 | 0.1226 |
| **LV end-diastolic volume BPS index BSA (ml/m^2^)** | 1.7 ± 0.3 | 1.5 ± 0.2 | 0.3779 |
| **LV end-systolic volume BPS (ml)** | 48 ± 14 | 33 ± 7 | 0.0604 |
| **LV ejection fraction BPS (%)** | 57 ± 3 | 56 ± 3 | 0.5547 |
| **Stroke volume (LVOT*VTI) (ml)** | 85 ± 21 | 59 ± 6 | **0.0260** |
| **Stroke volume index FFM (LVOT*VTI) (ml/kg)** | 1.3 ± 0.2 | 1.2 ± 0.2 | 0.4458 |
| **Stroke volume index BSA (LVOT*VTI) (ml/m^2^)** | 42 ± 8 | 34 ± 3 | 0.0624 |
| **LVOT diameter (mm)** | 22 ± 2 | 17 ± 1 | **0.0027** |
| **E/A Ratio** | 1.9 ± 0.4 | 1.9 ± 0.5 | 0.8845 |
| **Global Longitudinal Strain (%)** | -18.3 ± 1.2 | -18.1 ± 1.2 | 0.7860 |
| **Left Atria End-diastolic volume (ml)** | 57 ± 17 | 45 ± 14 | 0.2768 |
| **Right Atria End-diastolic volume (ml)** | 49 ± 13 | 36 ± 8 | 0.0984 |

**Supplemental table 2 -** Baseline left ventricular morphology and function in untrained male and female subjects. LV, Left ventricle; BSA, Body surface area; BPS, Biplane Simpsons method; LVOT, Left ventricular outflow track; E/A, early (E) and late (A) diastolic transmitral peak flow velocity; Values are mean ± SD.
